# Micropatterned Styrene–Butadiene–Styrene Thin Films Doped with Barium Titanate Nanoparticles: Effects on Myoblast Differentiation

**DOI:** 10.1021/acsbiomaterials.4c02468

**Published:** 2025-05-01

**Authors:** Leonardo Boccoli, Elena Drago, Andrea Cafarelli, Lorenzo Vannozzi, Angelo Sciullo, Federica Iberite, Sajedeh Kerdegari, Toshinori Fujie, Emanuele Gruppioni, Claudio Canale, Leonardo Ricotti

**Affiliations:** † The BioRobotics Institute, Scuola Superiore Sant’Anna, 56127 Pisa, Italy; ‡ Department of Excellence in Robotics & AI, Scuola Superiore Sant’Anna, 56127 Pisa, Italy; § Dipartimento di Fisica, Università di Genova, Via Dodecaneso 33, 16146 Genova, Italy; ∥ School of Life Science and Technology, Institute of Science Tokyo, 226-8501 Yokohama, Japan; ⊥ Research Center for Autonomous Systems Materialogy (ASMat), Institute of Integrated Research (IIR), Institute of Science Tokyo, 226-8501 Yokohama, Japan; # Centro Protesi INAIL, Istituto Nazionale per l’Assicurazione contro gli Infortuni sul Lavoro, 40054 Vigorso di Budrio, 40054 Bologna, Italy

**Keywords:** polymeric thin films, skeletal muscle tissue engineering, poly(styrene–butadiene–styrene), barium titanate nanoparticles, biohybrid

## Abstract

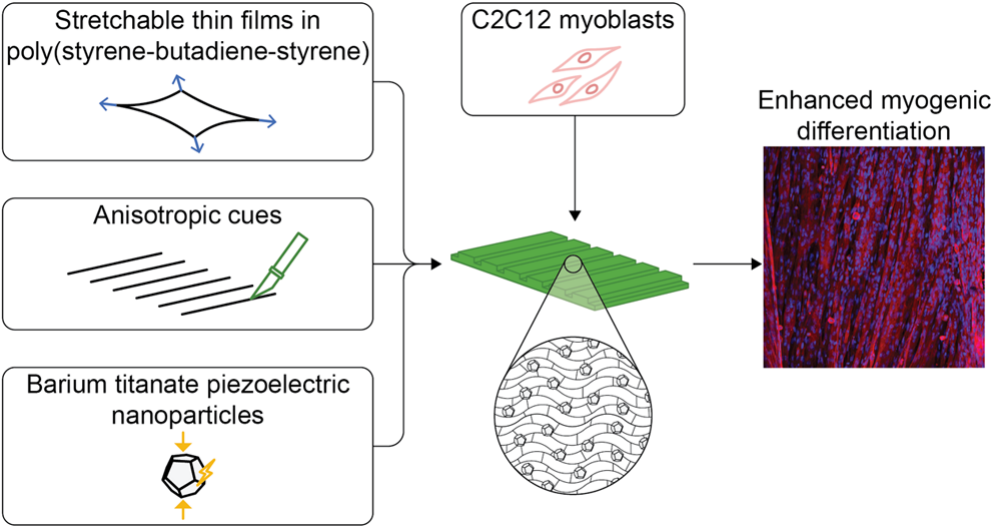

Biohybrid actuators exploit the contraction of biological components (muscle cells) to produce a force. In particular, bottom-up approaches use tissue engineering techniques, by coupling cells with a proper scaffold to obtain constructs undergoing contraction and guaranteeing actuation in biohybrid devices. However, the fabrication of actuators able to recapitulate the organization and maturity of native muscle is not trivial. In this field, quasi-two-dimensional (2D) substrates are raising interest due to their high surface/thickness ratio and the possibility of functionalizing their surface. In this work, we fabricated micropatterned thin films made of poly(styrene–butadiene–styrene) (SBS) doped with barium titanate nanoparticles (BTNPs) for fostering myogenic differentiation. We investigated material concentrations and fabrication process parameters to obtain thin microgrooved films with an average thickness below 1 μm, thus featured by a relatively low flexural rigidity and with an anisotropic topography to guide cell alignment and myotube formation. The embodiment of BTNPs did not significantly affect the film’s mechanical properties. Interestingly, the presence of BTNPs enhanced the expression of myogenic differentiation markers (i.e., MYH1, MYH4, MYH8, and ACTA1). The results show the promising potential of SBS thin films doped with BTNPs, opening avenues in the fields of biohybrid actuation and skeletal muscle tissue engineering.

## Introduction

Polymeric thin films are gaining growing attention for a plethora of applications in various fields, including biomedical engineering.^1^ Thin films are usually less than 1 mm thick substrates; when the film thickness is below 1 μm, they are called ultrathin films.^2^ This makes thin films quasi-two-dimensional (2D) substrates characterized by a high surface/thickness ratio and high versatility since their topographical and mechanical properties can be easily tuned. This enables several applications,^3^ including wound dressing,^4−7^ drug delivery,^8−10^ health monitoring,^11−14^ and tissue engineering.^15−18^

Thin films can be provided with topographical cues with a high degree of anisotropy, which is key to promote differentiation of cells toward a myogenic phenotype.^19−21^ Moreover, thin films show a flexural rigidity that is much smaller than that of the corresponding bulk structures made of the same material, thus resulting in enhanced flexibility. Among thermoplastic elastomers, poly(styrene–butadiene–styrene) (SBS) represents a promising material, as it exhibits rubber-like elasticity while maintaining the processability of plastic, making it an excellent candidate for creating flexible thin films with low flexural rigidity.^22^ These characteristics are especially advantageous for applications where mechanical compliance is critical such as biohybrid actuators. Importantly, SBS thin films have demonstrated the ability to support the differentiation of skeletal muscle cells into myotubes.^22^

Thin films can be easily functionalized from a chemical viewpoint and they can be provided with nanomaterials embedded in the polymeric matrix, offering the possibility to introduce additional helpful features.^23^ In fact, literature evidence that several types of nanoparticles have already been embedded in thin films, for example, iron oxide nanoparticles for enhancing temperature and humidity sensitivity of poly(3,4-ethylenedioxythiophene):poly(styrene sulfonate) (PEDOT:PSS) films,^24^ nanohydroxyapatite to induce osteogenicity of poly(ε-caprolactone) (PCL) membranes,^25^ or magnetic nanoparticles for influencing cell behavior when seeded on poly(lactic acid) (PLA) films.^26^

This is interesting especially if we consider piezoelectric nanomaterials, which have shown beneficial effects on myogenic differentiation, both alone or when activated through external mechanical stimulation.^27−29^ Piezoelectric nanoparticles have already been embedded in unpatterned thin films; in particular, zinc oxide (ZnO) nanoparticles embedded in PEG-*b*-PCL/PLLA films provided relatively high piezoelectric coefficients (peak values of 6 pm/V) and improved the myogenic differentiation of C2C12 myoblasts, increasing myotubes length.^27^ However, ZnO influence on cell adhesion and viability is still controversial.^30^ Moreover, the embedding of piezoelectric nanomaterials in patterned films and therefore the possible synergy between these two cues has not been investigated yet.

Among piezoelectric materials, barium titanate nanoparticles (BTNPs) have been explored due to their excellent cytocompatibility^31^ and high piezoelectric properties.^32,33^ These features make them especially interesting for engineering excitable tissues, such as skeletal muscle.

To the best of our knowledge, the culture of myoblasts on thin micro-grooved films doped with piezoelectric nanomaterials has not yet been explored. In this work, we investigate the influence of BTNPs on the chemical and physical properties of thin SBS films and how these micropatterned doped films can impact the myogenic differentiation of C2C12 cells.

## Experimental Section

### Fabrication

#### Mold Fabrication

Photolithographic masks with an anisotropic pattern were fabricated following the protocol reported by Hasebe et al.^22^ A poly(dimethylsiloxane) (PDMS) solution (Sylgard 184, Dow Corning Corporation, Midland, MI) with a monomer/curing agent 10:1 ratio was prepared, degassed using a vacuum pump, and poured over the previously prepared microgrooved silicon wafers inside glass Petri dishes (diameter: 9 cm). Petri dishes with PDMS were placed at 80 °C for 90 min to ensure complete PDMS cross-linking. Then, square micropatterned molds (2 × 2 cm) were cut using a scalpel to obtain PDMS masks.

#### Fabrication of Thin Films

**Figure 1:**
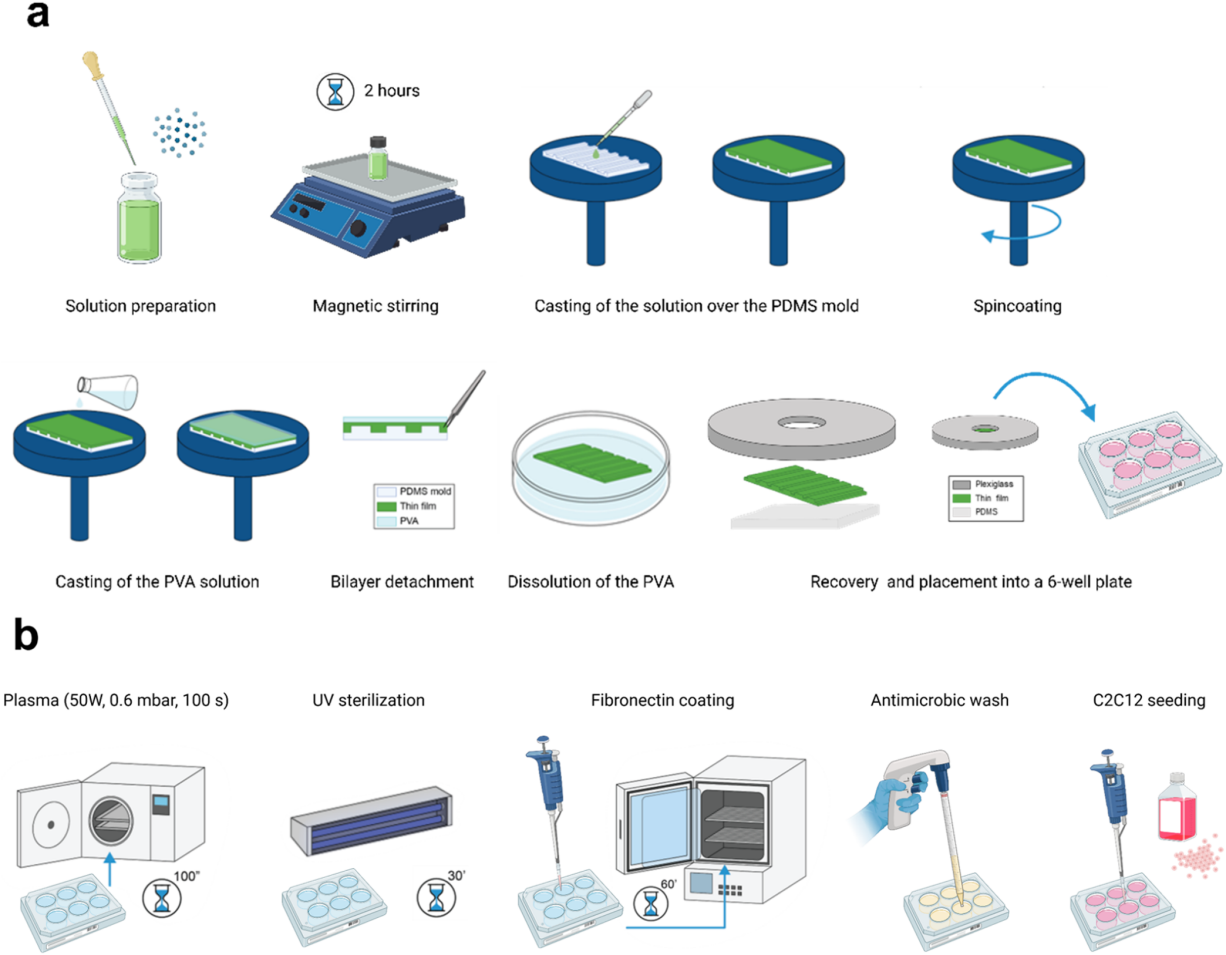
(a) Depiction of the different phases for fabricating micro-grooved doped styrene–butadiene–styrene thin films; (b) depiction of the procedure to prepare the thin films for cell culture experiments. Image created with BioRender, https://BioRender.com/t57u626.

Solutions of SBS (molecular weight: ∼140,000, 30 wt % styrene, Sigma-Aldrich, St. Louis, MO, 182877) in tetrahydrofuran (THF, Sigma-Aldrich, 401757) were prepared at concentrations of 20 and 40 mg/mL. BTNPs (diameter: 300 nm, GetNanoMaterials, Las Cruces, MN, 12047-27-7) were also added to the second solution (the chosen one after preliminary tests) at different concentrations (0.01, 0.1, 1% w/v). Also, nonpiezoelectric silica nanoparticles (diameter: 300 nm, Silica Nanospheres NanoXact Dried, nanoComposix, Inc., SISD300) were used at the concentration of 0.34% w/v. Solutions were mixed through magnetic stirring for 2 h and eventually submerged in an ultrasound water bath for up to 5 s to facilitate nanoparticle dispersion; then, solutions were poured on the PDMS molds. A spin-coating process (SPIN150, SPS Europe GmbH, Ingolstadt, Germany) was performed to produce thin films, with the rotational speed varied (1000, 2000, 4000 rpm) for 20 s. A 2% w/v solution of poly(vinyl alcohol) (PVA, molecular weight: 89,000–98,000, >99% hydrolyzed, Sigma-Aldrich, 341584) was cast over the films to form a water-soluble supporting layer, to facilitate the detachment of the film from the mold. Subsequently, the supporting layer was dissolved, putting the films in deionized water and thus obtaining self-standing SBS films. The procedure is depicted in Figure 1a.

### Characterization

#### Film Thickness Measurement

To discriminate the influence of SBS concentration and BTNP addition on film thickness, flat microfilms were produced by spin-coating on a glass. The films were scratched in their center using tweezers until they reached the underlying glass. A surface profilometer (KLA-Tencor Gambetti Kenologia SRL, Binasco, Italy) was used to measure the depth of the scratch, which in turn provided the film thickness. This measurement was repeated three times for each sample across three independent samples for each material formulation.

#### Mechanical and Topographical Characterization

Atomic force microscopy (AFM) analyses were carried out using an AFM microscope (Nanowizard IV, Bruker Co., Billerica, MA) integrated with an inverted optical microscope (DMi8, Leica Microsystems, Wetzlar, Germany) with 40× and 10× objectives. Samples were fixed on a glass slide via double-sided tape. A silicon nitride triangular cantilever (NPG, Bruker) with an elastic constant of 0.24 N/m, was used; the actual spring constant was determined through the thermal noise method.^34^ The nominal radius of the curvature of the tip was 20 nm.

The Quantitative Imaging (QI) mode was employed to acquire topographic and mechanical data simultaneously.^35^ A complete force–distance curve was obtained for each of the 256 × 256 pixels of the image, applying a force lower than 2 nN with a probe speed of 30 μm/s. AFM data were analyzed through the built-in Bruker software, fitting the curves with the use of the Hertz model for a sphere, considering the aforementioned radius of curvature, to finally obtain the Young’s modulus (*E*) map. The analysis was carried out on three independent samples per condition. The roughness was calculated from 1 μm × 1 μm size images acquired on three independent samples (at least three areas per sample), using the Gwyddion 2.62 free software.^36^ We calculated the mean roughness, defined in eq 1, as
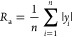
1where *n* is the total number of pixels, while *y*_*i*_ is the deviation of an image point from a mean line over the evaluation length.

Flexural rigidity correlates with Young’s modulus and thickness of a quasi-2D structure.^22^ It was calculated from the previously obtained data as in eq 2.
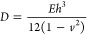
2being *E* the Young’s modulus, *h* the film thickness, and *v* the Poisson’s ratio (0.5 for elastomers^37^).

#### Film Manipulability

The manipulability of the microgrooved thin films, i.e., their ability not to wrinkle or self-fold in all fabrication process phases, was assessed qualitatively using a digital camera (EOS 90D, Canon, Inc., Tokyo, Japan) and an optical microscope (HRX-01, Hirox, Tokyo, Japan). The evaluation was also conducted quantitatively by assessing the ratio between the area of the PDMS mold and the area of the film after PVA dissolution and recovery on a glass slide. The analysis was carried out on three photos per sample over ten independent samples per condition, using the Fiji software.^38^

#### Morphological and Chemical Characterization

The surface topography of films was analyzed and reconstructed using an optical profilometer (DCM 3D, Leica) and the Gwyddion 2.62 software.^36^ Z-stacks (scan area of 1280 × 960 μm^2^) were acquired. Afterward, data were converted into 2D images provided with a height-related color scale. The Gwyddion software was used to measure groove width and height. The analysis was carried out three times per sample over three independent samples per condition.

The film surface was characterized by means of scanning electron microscopy (SEM) imaging. Prior to the analysis, samples were made conductive using a sputter coater (Quorum 150R ES, Quorum Technologies Ltd., Laughton, United Kingdom) applying a current of 30 mA for 90 s, thus covering them with a 10 nm-thick gold coating. Afterward, imaging was performed in secondary electron detection (SED) mode, using a dual beam (SEM-FIB) microscope (FEI Helios 600i Dual Beam SEM-FIB microscope, FEI Company, Hillsboro, OR) equipped with an energy-dispersive X-ray (EDX) detector (Bruker Quantax 200, Bruker Nano GmbH, Berlin, Germany), under high-vacuum conditions and setting a beam voltage of 15 kV and a current of 0.17 nA. EDX mapping and spectral acquisition were performed under the same conditions to evaluate the presence and distribution of BTNPs by detecting Ba and Ti atoms.

#### Thermal Characterization

The thermal behavior of the film formulations at different contents of BTNPs was investigated by differential scanning calorimetry (DSC) using a calorimeter (Star DSC 1, Mettler Toledo, Inc., Columbus, OH). Samples were prepared by pouring the SBS-BTNP solution into the sample holder and letting the solvent evaporate to get a final weight of ∼6 mg per sample. Each sample was heated from 25 to 100 °C with a heating rate of 10 °C/min, then kept at 100 °C for 2 min, cooled to −60 °C with a rate of 20 °C/min, then kept at −60 °C for 2 min, and finally heated to 100 °C with a rate of 10 °C/min. The material glass transition temperature was extrapolated from the cooling curve.

#### Piezoelectric Characterization

Piezoelectric properties were investigated by means of PFM (Icon Bruker AFM system), setting a scan frequency of 0.3 Hz and a scanning area of 1.5 × 1.5 μm^2^. A silicon probe with a Pt–Ir coating (SCM-PIT), a measured spring constant of 2.51 N/m, a resonant frequency of 67.4 kHz, and a deflection sensitivity of 112 nm/V was used. The amplitude and phase of the piezoelectric signals were acquired in the vertical direction via lock-in detection by applying to the tip an alternating current (ac) voltage of 1 V at a frequency of 60 kHz, which is outside the frequency resonance.

### Biological Characterization and Evaluation of Myogenic Differentiation

#### Setup and Sterilization for Cell Culture

Thin films for biological analyses were fabricated on a 2 × 2 cm PDMS mold and were irradiated for 30 min with UV light under a biological hood after the process (Safemate 1.2 Eco, BioAir SpA., Siziano, Italy). The PVA dissolution step was performed in autoclaved deionized water under a biological hood as well. To ease film handling in the subsequent experiments, the films were recovered through autoclaved 2 × 2 cm PDMS supports with the help of sterile tweezers (autoclaved at 121 °C, 1 atm, and 120 min). Films were left drying under the hood for 2 h, then placed in a 6-well plate, blocked to the bottom of the wells using circular plexiglass rings (Universal Laser Systems, VersaLaser VLS3.50), which exposed a central circular area of a diameter of 1 cm. These rings were previously sterilized through subsequent washes in deionized autoclaved water, 70% v/v ethanol (30 min), and again, with deionized autoclaved water.

After the setup assembly, films were plasma-treated to activate the SBS film surface. The oxygen plasma treatment (Colibr, Gambetti Kenologia SRL) was performed at 0.6 mbar with a power of 50 W for 100 s after 120 s of pressure stabilization. Then, samples were submerged in autoclaved deionized water, and further sterilization through UV light exposition (30 min) was done under the biological hood.

Sterile fibronectin from bovine plasma (Fn, Sigma-Aldrich, F1141) was diluted at a concentration of 50 μg/mL solution in Phosphate Buffer Saline with calcium and magnesium (PBS, Sigma-Aldrich, D1283); following the UV sterilization post plasma treatment, water was removed and the samples were covered in 3 mL of the above-mentioned Fn solution and incubated for 1 h at 37 °C in a 5% CO_2_ incubator, resulting in a final Fn coating density of 21.7 μg/cm^2^.

After incubation, the excess of the Fn solution was removed, and the films were incubated with 3 mL of PBS with the addition of 10% v/v penicillin/streptomycin (P/S, Sigma-Aldrich, P0781) and 0.1% v/v amphotericin B (Sigma-Aldrich, A2942) for 30 min at room temperature. Finally, the washing solution was removed, and the films were ready to use for cell seeding. The procedure for preparing thin films for cell culture experiments is depicted in Figure 1b.

#### Cell Culture

C2C12 murine skeletal myoblasts (ATCC, CRL-1772) were used as a skeletal muscle cell model. Cell culture was carried out at 37 °C in a CO_2_ incubator (5% CO_2_), using a growth medium (GM) composed of Dulbecco’s modified Eagle’s medium-high glucose (DMEM, Sigma-Aldrich, D5796) supplemented with 10% v/v fetal bovine serum (FBS, Sigma-Aldrich, F4135) and 1% P/S, and a differentiation medium (DM) composed of DMEM supplemented by 1% v/v P/S, 1% v/v FBS and 1% v/v Insulin-transferrin-sodium selenite media supplement (ITS, Sigma-Aldrich, I3146).

Cells were expanded until passage 14 and then seeded (GM0) at a cell density of 5 × 10^4^ cells/cm^2^ on previously prepared and sterilized micropatterned films with different nanoparticle concentrations (0.01, 0.1, and 1% w/v in the case of BTNPs and 0.34% w/v for silica nanoparticles, SNPs). Cells were cultured in GM for 4 days, changing the medium every other day. Then, GM was switched with DM to induce myogenic differentiation, and cells were cultured in DM for 6 days, replacing 2/3 of the medium with fresh DM every other day.

#### Cell Staining and Imaging

To visualize the differentiated myoblasts on the thin films, the cells were stained for F-actin on DM6. DM was removed, and samples were gently washed twice with PBS. Afterward, samples were fixed for 10 min with a 4% v/v paraformaldehyde (Thermo Scientific, Waltham, MA, 28908) solution in PBS. After two washes with PBS, cells were permeabilized with a 0.1% v/v solution of Triton X-100 (Sigma-Aldrich, T8787) in PBS (5 min). After two washes with PBS, the samples were then soaked in the staining solution for 1 h. The staining solution was composed of 2% v/v bovine serum albumin (BSA, PAN-Biotech GmbH, Aidenbach, Germany, P06-139310), 0.1% v/v Triton X-100 (Sigma-Aldrich, T8787), 0.1% v/v phalloidin–tetramethylrhodamine B isothiocyanate (Phalloidin-TRITC, Sigma-Aldrich, P1951), and 0.1% v/v Hoechst 33342 (Invitrogen, Waltham, MA, H1399) in PBS. Hoechst 33342 was added to the staining solution just for the final 30 min of incubation. Afterward, samples were washed twice with PBS. The entire procedure was carried out using 3 mL/well for each solution under a biological hood at room temperature.

Stained samples were imaged using a confocal inverted microscope (TCS SP8, Leica). The Fiji software^38^ was used to assess some parameters of myogenic differentiation, including the average width of myotubes, the fusion index (FI), and the percentage of area covered by myotubes. For each sample, three images were acquired. In particular, the FI was calculated using eq 3:
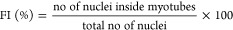
3

#### Gene Expression Analysis

Real-time quantitative reverse transcription polymerase chain reaction (qRT-PCR) was performed at two different time points (DM3 and DM6) to evaluate the expression of genes associated with myogenesis. For RNA extraction, the sample medium was removed, the samples were gently washed with PBS, and then RNA was extracted using ReliaPrep RNA Miniprep Systems (Promega, Madison, WI, Z6012) following the manufacturer’s protocol, and quantified with a Nanodrop 2000 (Thermo Scientific). A reverse transcription was performed with PrimeScript RT Master Mix (Takara Bio Inc., Kusatsu, Japan, RR036) according to the manufacturer’s instructions. Real-time qPCR was then performed with PowerUP SYBR Green Master Mix (Applied Biosystems, Waltham, MA, A25742) in a Rotor-Gene Q (Qiagen, Hilden, Germany), according to the manufacturer’s instructions. For relative gene expression quantification, GAPDH was used as a housekeeping gene and the 2^–ΔCt^ method was applied. A minimum of three independent samples were analyzed for each experimental group. All of the primers used are listed in Table S1.

### Statistical Analyses

All statistical analyses were performed using GraphPad Prism 8.0.2 (Dotmatics, Boston, MA). Experimental data were subjected to a Shapiro-Wilk normality test. Afterward, normally distributed data were analyzed by two-way analysis of variance (ANOVA), followed by Tukey’s post hoc testing for multiple comparisons. Otherwise, non-normally distributed data were analyzed with the Kruskal–Wallis test, followed by Dunn post hoc testing for multiple comparisons. Normally distributed results were expressed as mean values ± standard deviations and plotted as histograms; non-normally distributed data were shown with either box plots (median value, 25th and 75th quartile ± max/min whiskers) or violin plots. Outliers were filtered through ROUT analysis (*Q* = 1%). The significance threshold was set at 5%, computing a two-tailed *p*-value. *p*-values of 0.05, 0.01, 0.001, and 0.0001 were identified as *, **, ***, and ****, respectively.

## Results

### Thin SBS Film Fabrication and Characterization

Two SBS concentrations (20 and 40 mg/mL) and three different spin-coating speeds (1000, 2000, and 4000 rpm) were tested in the first phase. Film thickness was evaluated to select the most appropriate spin-coating speed, while Young’s modulus and manipulability were analyzed to choose the SBS concentration that allowed the testing of manipulable and stable freestanding thin films.

**Figure 2:**
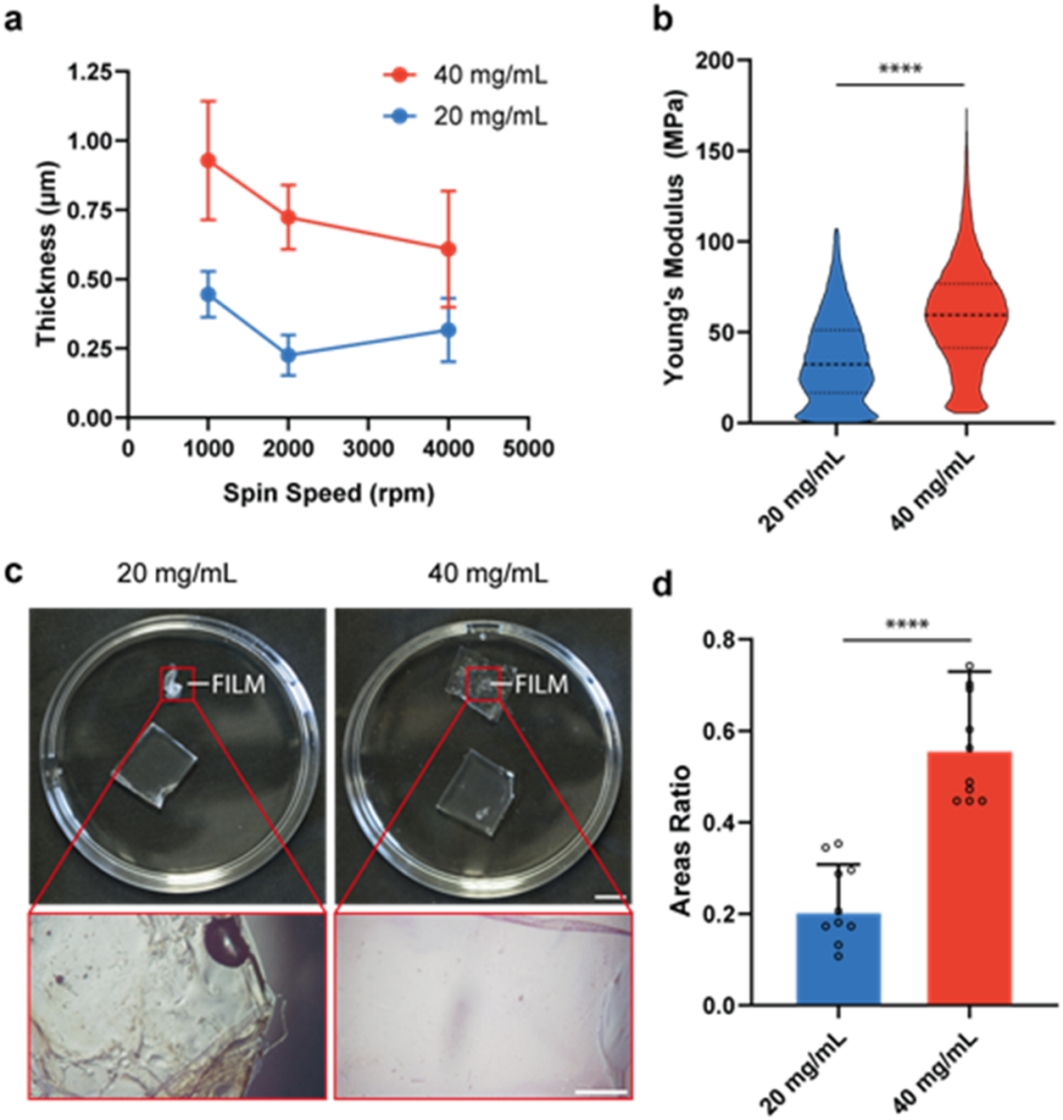
Characterization of thin films. (a) Thickness of SBS films by varying SBS concentration and spin-coating speed; (b) film Young’s modulus by varying SBS concentration, data filtered for outliers; (c) images of freestanding films with different SBS concentrations after PVA dissolution (scale bars: 1 cm for top images, 1 mm for close-ups - magnification 100×); (d) film/mold area ratio by varying SBS concentration; overlaid scattered dots in each experimental group represent a biological replicate (*n* = 10). Films in (b–d) were obtained by setting a spin-coating speed of 2000 rpm. **** = *p* < 0.0001.

Figure 2a shows the film thicknesses obtained. The average values were <1 μm in all of the tested conditions. Based on these results, and in accordance with the work of Hasebe et al.,^22^ a speed of 2000 rpm was selected, corresponding to an average thickness of 0.226 μm for 20 mg/mL and of 0.732 μm for 40 mg/mL. Such thickness values, in fact, were proven as appropriate to favor the contraction of myotubes cultured on the film surface.

The Young’s moduli of the films obtained using different SBS concentrations are plotted in Figure 2b. The analysis showed significantly different moduli for the two concentrations. In particular, the average *E* was 35.24 MPa for 20 mg/mL, and 60.39 MPa for 40 mg/mL.

Although the first condition (20 mg/mL) allowed the minimization of Young’s modulus, which would favor film contraction, the handling assessment (Figure 2c,d) revealed the difficulty in adequately using films obtained with this concentration: in fact, films tended to wrinkle and fold, resulting in an average area ratio of 20%. The SBS concentration of 40 mg/mL resulted in much more stable films with an average area ratio of 56%. This concentration was selected for subsequent experiments.

### Nanoparticles Embedding in Thin SBS Films

Thickness and manipulability were also evaluated on thin films produced with the previously optimized parameters (40 mg/mL, 2000 rpm) by varying the BTNPs concentration.

**Figure 3:**
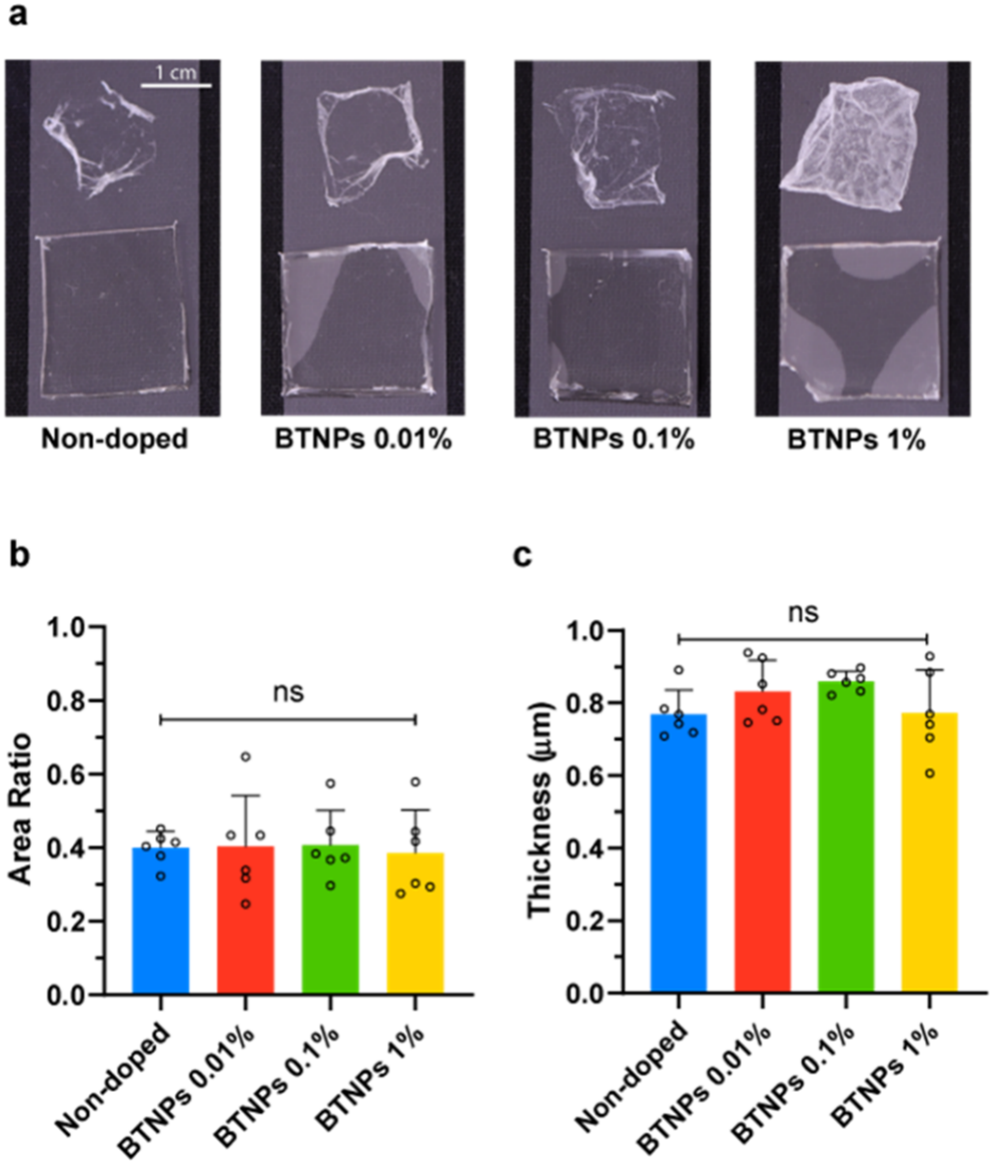
Characterization of doped films in terms of thickness and manipulability. (a) Images of films (SBS 40 mg/mL) with different BTNP concentrations after PVA dissolution (scale bar 1 cm); (b) film/mold area ratio by varying the BTNP concentration to quantify manipulability; overlaid scattered dots in each experimental group represent biological replicates (*n* = 6). (c) Film thickness by varying BTNP concentration; overlaid scattered dots in each experimental group represent biological replicates (*n* = 6), each derived from the average of three different measures per film.

Figure 3a,b shows the results concerning the manipulability of the doped films. The inclusion of BTNPs did not significantly enhance the film/mold area ratio. Results concerning the thickness are shown in Figure 3c. No significant difference in thickness was observed among the groups; the average thickness remained below 1 μm in all cases.

### Analysis of Film Morphology

**Figure 4:**
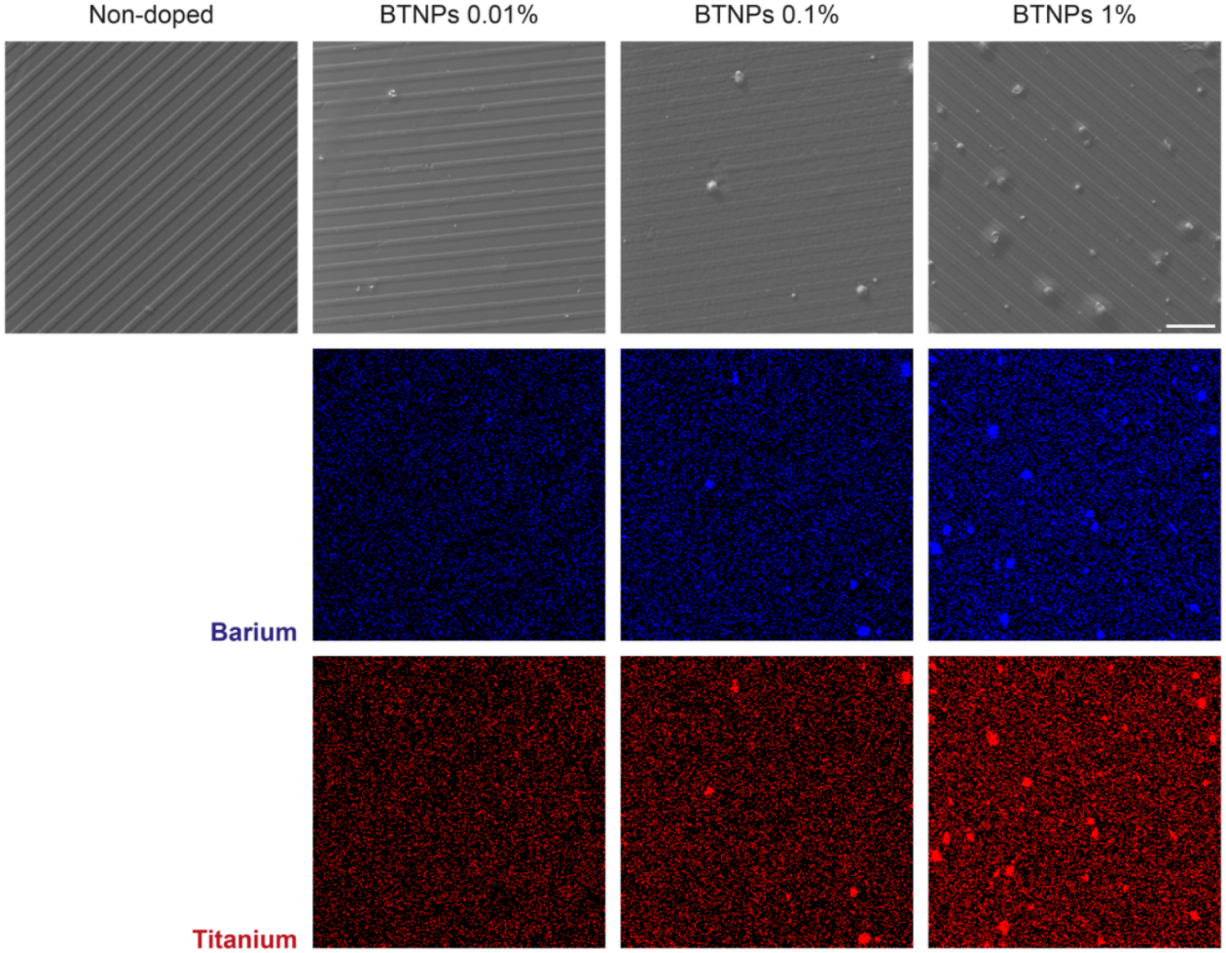
SEM images (top) and EDX maps of Ba (center) and Ti (bottom) for SBS films with different BTNP concentrations. Magnification: 880×, scale bar = 50 μm.

Optical profilometer analyses (Figure S1) were used to investigate the surface topography. Thin films presented the desired micropatterning in all conditions and a groove height (∼1 μm) and width (∼10 μm) coherent with the dimensions of the photolithographic mask used for PDMS mold production (Figure S2). SEM imaging (Figure 4) further confirmed the presence of the microgrooves, and the EDX analysis highlighted the presence of Ba and Ti, suggesting a uniform distribution of BTNPs within the polymeric matrix and an increasing Ba and Ti content over the BTNP concentration. The presence of the polymeric material (i.e., SBS) within the organic solvent increased the dispersibility of BTNPs, as the polymer likely acted as a surfactant, wrapping the nanoparticles and reducing their tendency to form clusters.

**Figure 5:**
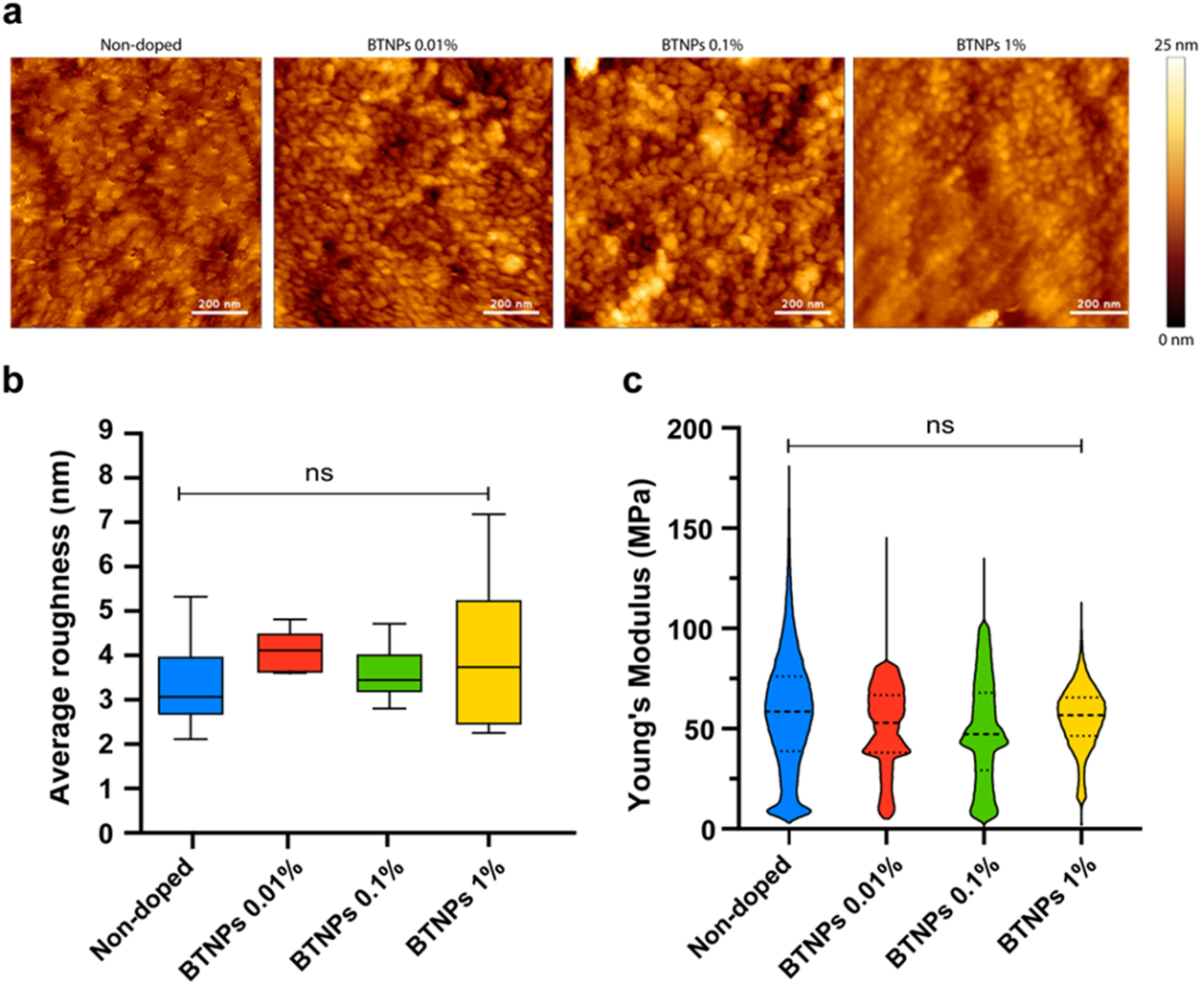
AFM characterization of the doped SBS films. (a) Representative high-magnification AFM images acquired on the surface of the groove top and groove bottom for SBS films with different BTNP concentrations. (b) Average roughness of the thin films. A minimum of 45 areas have been analyzed, per each sample type. (c) Young’s modulus of SBS films with different BTNP concentrations, data filtered for outliers.

AFM scans allowed a refined analysis of film surface morphology and mechanical properties (Figures 5, S3, and Table S2).

Results showed that the concentrations of nanoparticles did not significantly alter the surface roughness (Figure 5a,b). AFM nanoindenting analyses also revealed no significant influence of BTNPs on the film stiffness (Figure 5c), even at high concentrations. We noticed the presence of a higher number of points with outliers, suggesting high stiffness in the sample at the BTNP concentration of 1%. We treated these data as outliers, rejecting them. Probably, such stiffer points were associated with the presence of nanoparticles exposed on the sample surface. Although their presence cannot be directly proven by AFM imaging, it can be observed in SEM images, as shown in Figure 4. A second graph showing stiffness, considering all data, is reported in Figure S4.

### Thermal Characterization

DSC analyses (Figure S5) underlined the presence of a glass transition temperature of SBS at 96–97 °C in all samples due to its amorphous nature. The addition of nanoparticles did not affect this property. No melting point was found in the temperature range of the heating curve. This suggests that the thermal properties of the films remained stable during the culture period. Indeed, this was confirmed by morphological analyses carried out before and after keeping the samples in cell culture conditions (Figure S6 and Table S3).

### Biological Results

To investigate the influence of micropatterning and piezoelectric nanoparticles embedding on myotube formation and alignment, C2C12 cells were seeded onto nondoped and doped films with different concentrations of BTNPs and allowed to grow for 4 days and then differentiate for 6 days.

**Figure 6:**
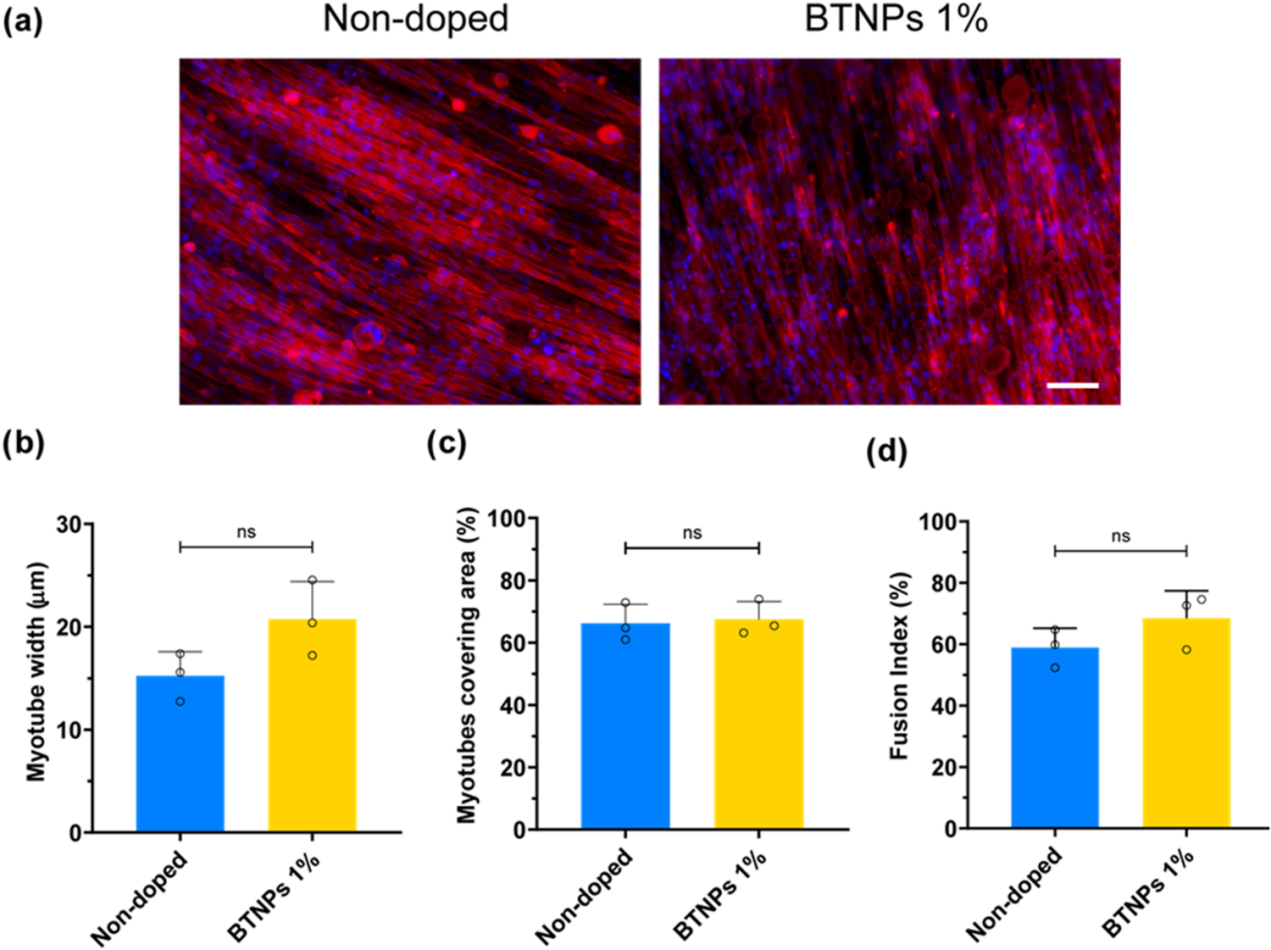
C2C12 differentiation on the doped and nondoped SBS films. (a) Staining of F-actin (red) and nuclei (blue) of C2C12 cells seeded on nondoped films and films doped with 1% BTNPs, on the 6th day in differention medium (DM6). Scale bar = 100 μm; (b) quantification of myotube width; (c) area covered by myotube; (d) fusion index. Overlaid scattered dots in each experimental group represent biological replicates (*n* = 3), each derived from the average of three technical replicates.

F-actin and its nuclei are shown in Figure 6. The myotubes showed a preferential orientation axis over the film surface, fostered by the anisotropic micropatterning. The nanoparticles in the substrates did not significantly influence myotube morphology: the mean values of myotube width and fusion index showed an increase in the thin films doped with 1% BTNPs with respect to the nondoped controls (20.72 ± 3.67 vs 15.24 ± 2.33 μm for myotube width and 68.48 ± 8.92 vs 58.62 ± 6.23% for fusion index), but these differences were not statistically significant.

**Figure 7:**
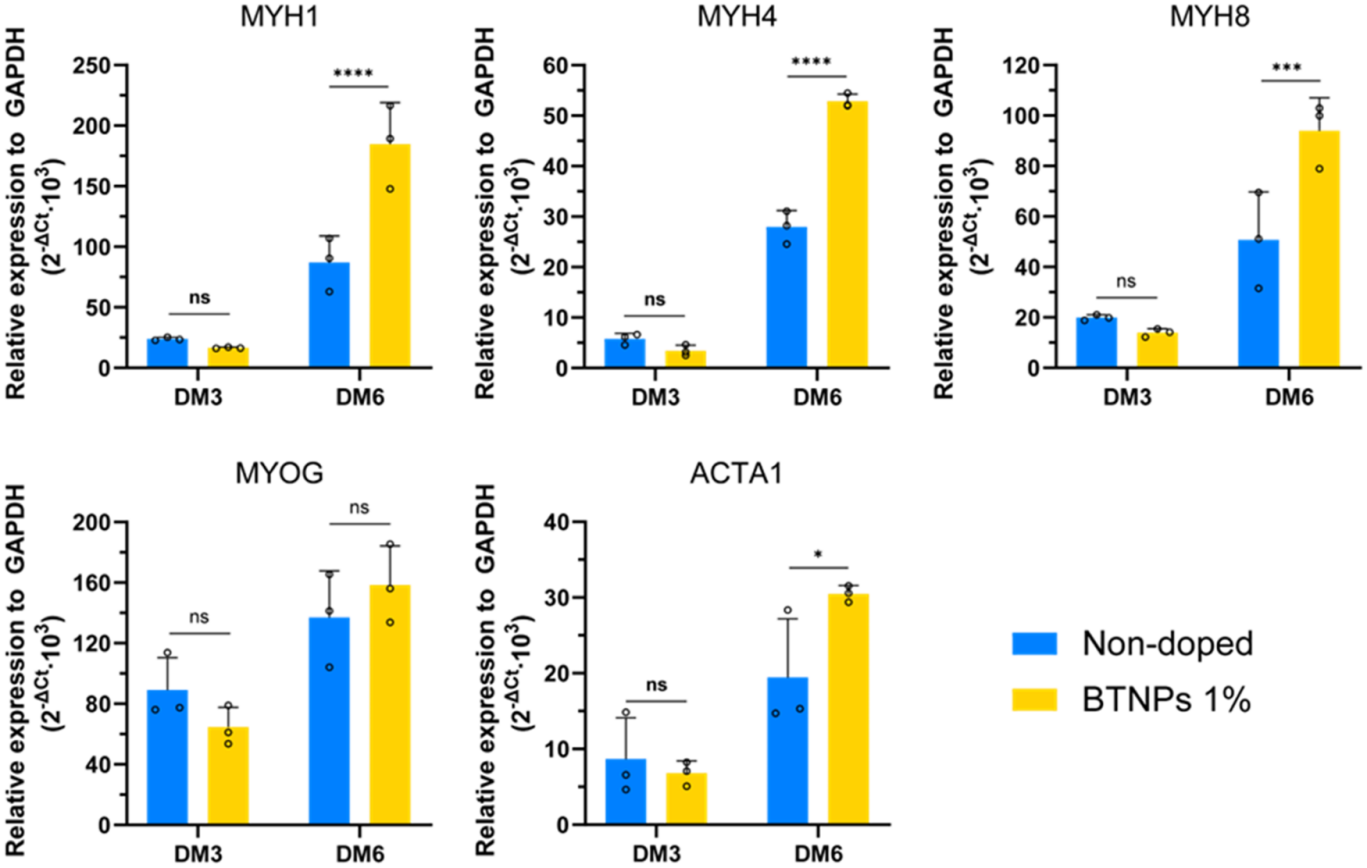
Gene expression analyses on myoblasts differentiated on nondoped and doped films, on days 3 and 6 of differentiation. DM: differentiation medium. * = *p* < 0.05, *** = *p* < 0.001, **** = *p* < 0.0001. Overlaid scattered dots in each experimental group represent biological replicates (*n* = 3), each derived from the average of three technical replicates.

Results of gene expression analyses are shown in Figure 7. Interestingly, the presence of 1% BTNPs boosted the expression of all genes analyzed at DM6, except for MYOG, with respect to nondoped controls. Notably, MYH1 and MYH8 both displayed a 1.7-fold increase in their median values, while MYH4 exhibited a 1.8-fold increase. Meanwhile, ACTA1 also demonstrated an appreciable increase, reaching a 1.3-fold enhancement.

It can be observed that the expression for these genes followed an increasing trend in doped films from DM3 to DM6, while it remained constant during the differentiation process for nondoped films.

## Discussion

The interesting properties of thin films are mostly due to their thickness, which can be carefully tuned by acting on the fabrication process parameters. In fact, micropatterned SBS thin film thickness depends on the SBS concentration in the starting polymeric solution.^22^ Moreover, spin-coating speed is another parameter that can influence film thickness.^55^ These parameters can directly affect the mechanical characteristics of the films, namely, their elastic modulus and, consequently, their flexural rigidity.^22^ For these reasons, we optimized the fabrication process parameters necessary to minimize the thickness and Young’s modulus, to ensure reliable handling of films in all of the fabrication phases and to allow the possible contraction of cultured myotubes on the surface.

No significant differences in film thickness between the three spin-coating speeds evaluated were observed. A spin-coating speed of 2000 rpm had already been used in literature for micropatterned thin film fabrication,^22,27^ obtaining thickness values similar to the ones achieved in this work.

SBS concentration plays a relevant role in the control of the thickness and mechanical properties of thin films. A SBS concentration of 40 mg/mL resulted in about a 2-fold increase in film thickness and Young’s modulus with respect to 20 mg/mL. Previous works on SBS 2D films^22,39^ highlight similar considerations on the dependency of mechanical properties on polymer concentration, although a different evaluation method was considered: such works used tensile or bulge tests, while we performed nanoindenting analyses. Films fabricated using a concentration of 20 mg/mL showed smaller flexural rigidity; however, our results on manipulability highlighted that these samples were challenging to manage, due to their tendency to self-fold after PVA dissolution in water. Therefore, the selected concentration of SBS for subsequent experiments was 40 mg/mL.

Using the above-mentioned concentration and speed, the obtained values of thickness (around 0.8 μm) and Young’s modulus (around 60 MPa) are comparable to the ones obtained in previous investigations on SBS films, in which *E* ranges from 30 to approximately 70 MPa.^22,39^ However, the elastic moduli obtained are 3 orders of magnitude higher than the ones of natural skeletal muscle tissue,^40,41^ and the target value for optimal myogenic differentiation and contraction of substrates for skeletal muscle tissue engineering, generally identified in 10–15 kPa.^42,43^ Nevertheless, thin films show enhanced flexibility with respect to corresponding three-dimensional (3D) structures due to their high surface/thickness ratio. A work from our group highlighted the role of flexural rigidity in predicting the ability of thin films to undergo contraction when myotubes exert a force on them.^44^ The obtained average value of flexural rigidity, considering the values of *E* and thickness of the films obtained with the set parameters, is 3.43 × 10^–12^ Nm. This flexural rigidity is considered in the range identified by previous works.^22,44^ It may ensure both the structural stability of the film and the contractility of the film when subjected to forces exerted by cultured myotubes.^45^

The addition of BTNPs did not influence either the thickness or elastic modulus. The inclusion of nanoparticles has been proven to affect the thickness of thin films,^24,26^ but this has been obtained for thickness values smaller than the ones achieved in our case. Also, in other words, the embodiment of piezoelectric nanoparticles in polymeric films produced an increase in the elastic modulus.^27,28^ Our results seem in contrast with such evidence. This could be due to the specific SBS properties: SBS is an elastomer, while the evidence reported in the state-of-the-art concerned thermoplastic polymers (PEDOT:PSS,^24^ PLA,^26^ PEG-*b*-PCL,^27^ PLLA,^27^ PLGA^28^).

Anisotropic surfaces, characterized by physical properties that vary with direction, have been shown to significantly influence cell behavior, particularly in directing cell orientation and differentiation, a particularly relevant aspect in designing tissue engineering scaffolds for skeletal muscle regeneration. Anisotropy promotes cell orientation and differentiation, particularly for skeletal muscle precursors.^15,22,46−48^ This is because the inherent directional alignment present in these materials, whether it be microchannels, ridges, or aligned fibers, provides contact guidance cues to the cells. These cues influence cell adhesion, morphology, migration, and alignment, ultimately leading to enhanced differentiation. For instance, microchannels ranging from 2 to 30 μm have been shown to align myotubes along the grooves and induce the expression of myogenic markers.^22,49^ The depth of these grooves is a crucial factor, influencing cell elongation and the degree of alignment.^50^ As groove depth increases, cell spreading is often inhibited, and proliferation can be retarded.^51^ However, this is often accompanied by improved cellular alignment and elongation along the anisotropic direction.^52^ This response to depth highlights the ability of cells, particularly myoblasts, to sense and respond not only to surface topographical cues but also to three-dimensional features of their microenvironment.

The micro-grooved morphology achieved in our study aligns with these findings: optical profilometry showed the presence of the desired micro-grooved morphology on the film surface, reflecting the pattern on the PDMS molds. The average groove width and height resulted in around 9.5 and slightly less than 1.2 μm, respectively. The slight difference in the groove dimensions from the photolithographic mask pattern (10 and 1 μm respectively) could be due to plastic deformation during the detachment of films from the molds, already described by Hasebe et al.^22^ The BTNPs embodiment did not affect micropatterning in terms of the average groove width and height.

SEM analyses confirmed the aforementioned results about the surface topography of the films. In addition, they qualitatively showed the homogeneous dispersion of BTNPs in the films. This result is in agreement with previous works using piezoelectric nanoparticles for thin film production.^27^ EDX analyses highlighted the presence of Ba and Ti, evidencing the nanoparticle homogeneous distribution.

Thermal analyses underlined a glass transition of SBS at 96–97 °C, as already found in literature,^53^ with a negligible influence due to the BTNPs presence. This result is probably due to the sharp glass transition of styrene blocks.^53,54^

Biological characterization on both nondoped and doped (BTNPs 1% w/v) films showed a high differentiation level, as suggested by cell morphology in fluorescent staining imaging and fusion index values. Myoblasts efficiently fused into aligned myotubes, having a preferential orientation provided by the microgroove anisotropy. These results confirmed the usefulness of microgrooved patterning in directing myotubes formation and unidirectional alignment.^55^

Compared with the control, the samples doped with BTNPs at 1% (w/v) showed myotubes with a slightly larger width and fusion index, even though the differences were not statistically significant. Nonetheless, gene expression analyses revealed the key role of BTNPs in promoting myogenic differentiation. In particular, the BTNPs embedded in the film matrix induced an overexpression of myosin heavy chain-related genes after 6 days of differentiation. These markers, coding for the myosin proteins in sarcomeres,^56^ are linked to the primary (MYH8) and the secondary myogenesis (MYH2 and MYH1) of skeletal muscle in mice.^57^ BTNPs also upregulated the expression of ACTA1, a gene involved in the production of α-skeletal actin. This protein is ubiquitous in all cells but is especially related to sarcomere formation and functions in skeletal muscle.^58^ All of these genes are typically upregulated in C2C12 cells that are undergoing the process of differentiation into more mature myotubes,^59^ suggesting a high maturation of C2C12 cells. Lastly, the BTNP embodiment had no effects on the expression of MYOG, which is an early differentiation marker. The observed myogenesis enhancement appeared to be significantly influenced by the piezoelectric properties introduced by the BTNPs. These properties could be leveraged through subtle mechanical interactions of migrating cells or early myotubes, showing spontaneous contractile activity, creating a supportive microenvironment (including electrical stimuli) that promoted skeletal muscle differentiation. Additionally, the effect of nanometric surface roughness on myogenic differentiation has not been unanimously determined. In fact, a study by Hu and colleagues on the differentiation of human mesenchymal stem cells revealed a better behavior of smooth substrates in directing stem cells toward myogenesis.^60^ At the same time, several works state the positive contribution of roughness on the maturation of myotubes;^28,61^ higher nanoroughness is, in fact, meant to lead to higher protein adsorption and cell adhesion.^62,63^ However, we did not observe a difference in the surface roughness after embedding nanoparticles in the films at different concentrations. This allows us to exclude roughness as one of the main factors responsible for myogenic differentiation in the doped substrates.

To confirm that the differentiation enhancement was due to the piezoelectricity of BTNPs, we also cultured cells on thin films doped with nonpiezoelectric SNPs of the same size as BTNPs. To account for the density differences (δ_SNPs_ = 2.2 g/cm^3^, δ_BTNPs_ = 6.1 g/cm^3^),^64,65^ we used a 0.34% w/v concentration of SNPs to keep the same volumetric fraction of the nanoparticles as in the 1% w/v BTNP-doped films, equal to 0.17% (v/v).

Results show that BTNPs had a *d*_33_ coefficient equal to 85.2 ± 8.4 pm/V, which is in line with the values reported in the scientific literature.^32,66^ On the other hand, SNPs, used as nonpiezoelectric controls, exhibited low piezoelectricity (3.8 ± 1.9 pm/V). Figure S7 shows representative topographic images of the two types of nanoparticles and an example of the output of the measurement performed. In Figure S8, the difference in the hysteresis between the two materials is also evident, as BTNPs exhibit a clear “butterfly effect”.

The addition of either BTNPs or SNPs to thin films did not result in any alteration in the substrate surface roughness; furthermore, Young’s modulus was not influenced by the presence of both nanoparticle types within the polymeric matrix (Figure S9). Nevertheless, both nondoped and SNP-doped films exhibited comparable differentiation levels, which were lower than those observed in the 1% w/v BTNP group. Although this enhancement is not evident in terms of myotube features (Figures S10 and S11), gene expression analyses revealed substantial differences in the expression of myogenic markers between the 1% BTNP-doped group and the other two groups (nondoped substrates and SNP-doped ones, Figure S12).

Since no differences were observed in the properties of BTNP-doped and SNP-doped films (Figure S13), except from piezoelectricity, we conclude that the enhancement of myotube differentiation in BTNP-doped substrates is primarily due to the piezoelectric properties of the embedded nanoparticles.

BTNPs’ piezoelectricity could produce small electrical stimuli during the differentiation process, caused by the small local deformation of BTNPs following cell migration and attachment. Electrical stimulation, indeed, is known to have high potential in boosting maturation of electroactive tissues, such as the skeletal muscle.^19^ The role of BTNPs internalized in cells to foster myogenic differentiation has already been demonstrated.^29,48,67^ The possibility of embedding piezoelectric materials in matrices has also been explored, exploiting their direct or indirect piezoelectric effect upon mechanical or electrical stimulation.^68,69^ To the best of our knowledge, the effect of piezoelectric nanomaterials not activated by external stimuli but simply embedded in a polymeric matrix, coupled with topographical cues given by films’ micropatterning, has not been investigated yet. Previous works underlined the benefits of piezoelectric nanoparticles (in particular, ZnO) embedded in biodegradable substrates on myogenesis;^27,70^ however, not in combination with anisotropic topographical cues. Similar considerations apply to studies in which poled piezoelectric poly(vinylidene fluoride)^71,72^ or poly-3-hydroxybutyrate/poly-β-alanine^73^ were used.

## Conclusions

In this work, micropatterned thin films made of SBS and doped with piezoelectric BTNPs were investigated in terms of chemicophysical properties and ability to foster myoblast differentiation. SBS films with tailored surface topography and high manipulability were produced, featuring a thickness smaller than 1 μm. The embodiment of BTNPs did not significantly alter the physical, mechanical, and morphological features of thin films, even at the highest concentration used. Interestingly, the presence of piezoelectric nanoparticles fostered myogenic differentiation, as demonstrated by the enhanced expression of myogenic differentiation markers. Our results outline the usefulness of piezoelectric nanomaterials as a dopant agent of polymeric matrices to boost skeletal muscle differentiation, excluding, at the same time, the possible influence of the doping process on other film properties. Thin films based on this paradigm can find application in the field of skeletal muscle tissue engineering, particularly in the perspective of building tissue-engineered muscle grafts stacking multiple films, overcoming the need for vascularization techniques.^74^ In this context, the beneficial effects guaranteed by the inclusion of piezoelectric nanoparticles, which concretized in higher expression of genes key for myogenesis in this in vitro study, could turn into further beneficial effects in a longer time frame, especially in an in vivo scenario. Indeed, the indirect electrical stimulation provided by the piezoelectric scaffold (that could be further empowered by providing external ultrasound stimulation) could trigger additional beneficial effects on vasculogenesis,^75^ neural regeneration,^76^ and other phenomena that could not be investigated in this work.

## Supporting Information Available

Characterization of film surface topography through optical profilometry (Figure S1); surface micropatterning characterization (Figure S2); microgrooves thickness characterization through AFM analysis (Table S2); AFM imaging showing the micropatterned topography of SBS films (Figure S3); Young’s modulus of SBS films with different BTNP concentrations and SNP (Figure S4); thermal characterization of SBS films with different BTNP concentrations (Figure S5); SEM images for SBS films with and without 1% BTNPs and 0.34% SNPs (Figure S6); grooves’ width before and after being maintained for 10 days in cell culture conditions (Table S3); representative images of the topography of BTNPs and SNPs (Figure S7); comparison between the amplitude and phase signals of for silica (SNPs) and barium titanate (BTNPs) nanoparticles (Figure S8); AFM characterization of the doped SBS films (Figure S9); evaluation of myotube formation on SBS films with different BTNP concentrations (Figure S10); quantitative myotube analysis derived from immunofluorescence images (Figure S11); and gene expression analyses on myoblasts differentiated on nondoped and doped films, on days 3 and 6 of differentiation (Figure S12); characterization of doped films in terms of thickness and manipulability (Figure S13) (PDF)
Fabrication procedure for obtaining the SBS thin films (Video S1) (MP4)
