# Supplementary material for: Micropatterned Styrene–Butadiene–Styrene Thin Films Doped with Barium Titanate Nanoparticles: Effects on Myoblast Differentiation
Source: ACS Biomater Sci Eng. 2025 May 1;11(5):2910–21. doi: 10.1021/acsbiomaterials.4c02468 (PMC12076278; doi:10.1021/acsbiomaterials.4c02468)
Supplement: Supplementary file 1 — ab4c02468_si_001.pdf [file ab4c02468_si_001.pdf]

Supporting information for publication

Micropatterned styrene-butadiene-styrene thin films doped with barium titanate nanoparticles: effects on myoblast differentiation

Leonardo Boccoli[1,2,‡], *Elena Drago*[1,2,‡], *Andrea Cafarelli*[1,2,‡], *Lorenzo Vannozzi*[1,2,‡], *Angelo Sciullo*[1,2,‡], *Federica Iberite*[1,2], *Sajedeh Kerdegari*[3], *Toshinori Fujie*[4,5], *Emanuele Gruppioni*[6], *Claudio Canale*[3], *Leonardo Ricotti*[1,2,*]

[1] The BioRobotics Institute, Scuola Superiore Sant'Anna, 56127 Pisa, Italy

[2] Department of Excellence in Robotics & AI, Scuola Superiore Sant'Anna, 56127 Pisa, Italy

[3] Dipartimento di Fisica, Università di Genova, Via Dodecaneso 33, Genova, 16146, Italy

[4] School of Life Science and Technology, Institute of Science Tokyo, 226-8501 Yokohama, Japan

[5] Research Center for Autonomous Systems Materialogy (ASMat), Institute of Integrated Research (IIR), Institute of Science Tokyo, 226-8501 Yokohama, Japan

[6] Centro Protesi INAIL, Istituto Nazionale per l'Assicurazione contro gli Infortuni sul Lavoro, 40054 Vigorso di Budrio (Bologna), Italy

[*] Author to whom correspondence should be addressed. E-mail: leonardo.ricotti@santannapisa.it

[‡] Both authors equally contributed to this work

Number of pages: 10

Number of tables: 3

Number of figures: 13

Gene targeted	Primer sequence (5’ – 3’)
**GAPDH**	Fw: CGACTTCAACAGCAACTCCCACTCTTC<br>Rv: TGGGTGGTCCAGGGTTTCTTACTCCTT
**ACTA1**	Fw: ACCATCGGCAATGAGCGTT<br>Rv: GCTGTTGTAGGTGGTCTCATGG
**MYOG**	Fw: CCCATGGTGCCCAGTGAA<br>Rv: GCAGATTGTGGGCGTCTGTA
**MYH1**	Fw: GAGGGACAGTTCATCGATAGCAA<br>Rv: GGGCCAACTTGTCATCTCTCAT
**MYH4**	Fw: GAAGGAGGGCATTGATTGG<br>Rv: TGAAGGAGGTGTCTGTCG
**MYH8**	Fw: ACTGAGGAAGACCGCAAGAA<br>Rv: CAGGTTGGCATTGGATTGTTC

Table S1: Mouse-specific primer sequences used for real-time RT-qPCR analyses in the myoblast differentiation experiment on the SBS films.

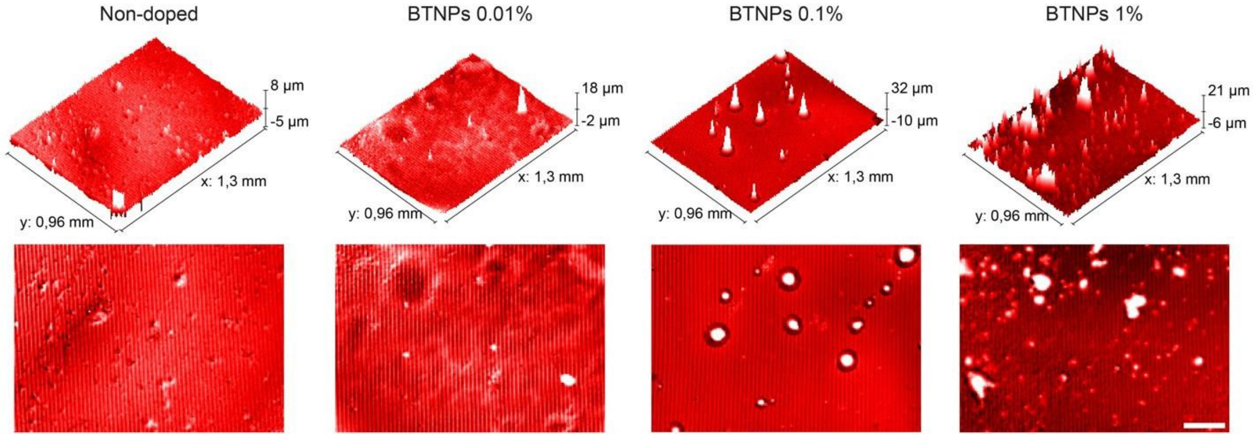

Figure S1: Characterization of film surface topography through optical profilometry. Representative 3D maps (top) and 2D images (bottom) are shown for SBS films with different BTNP concentrations. Scale bar = 200 µm.

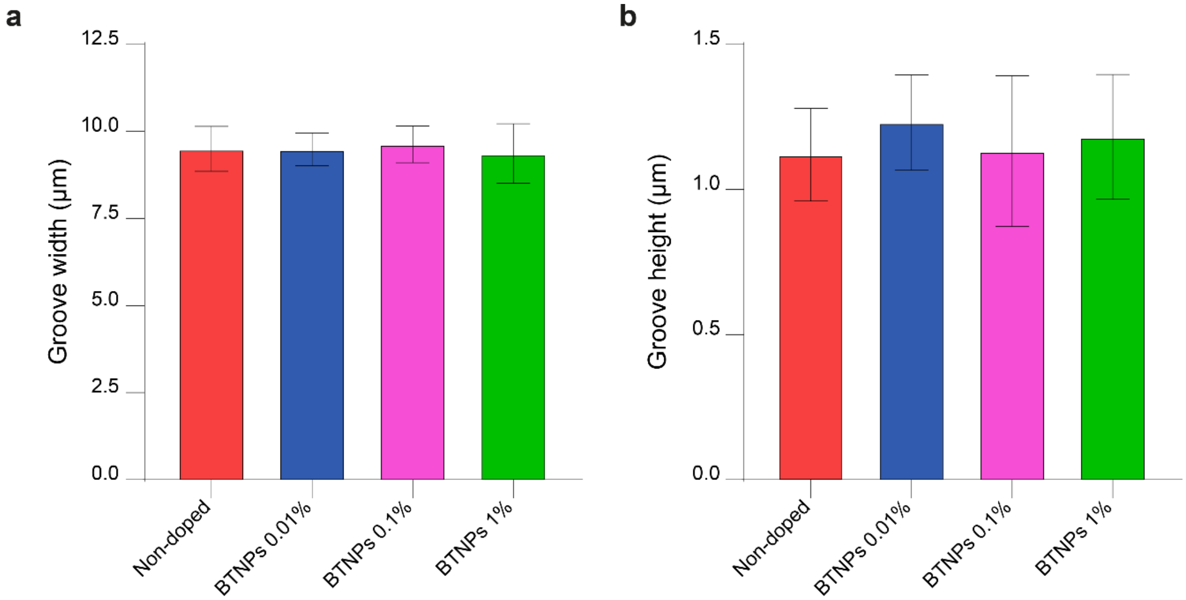

Figure S2: Surface micropatterning characterization. (a) Groove width; (b) groove height. Both indexes were measured on images derived from optical profilometer analyses, for SBS films with different BTNP concentrations.

Sample	Average thickness (µm)	Standard deviation (µm)
**Non-doped**	1.04	0.08
**BTNP 0.01%**	0.93	0.10
**BTNP 0.1%**	0.92	0.07
**BTNP 1%**	1.0	0.20

Table S2: Microgrooves thickness characterization through AFM analysis.

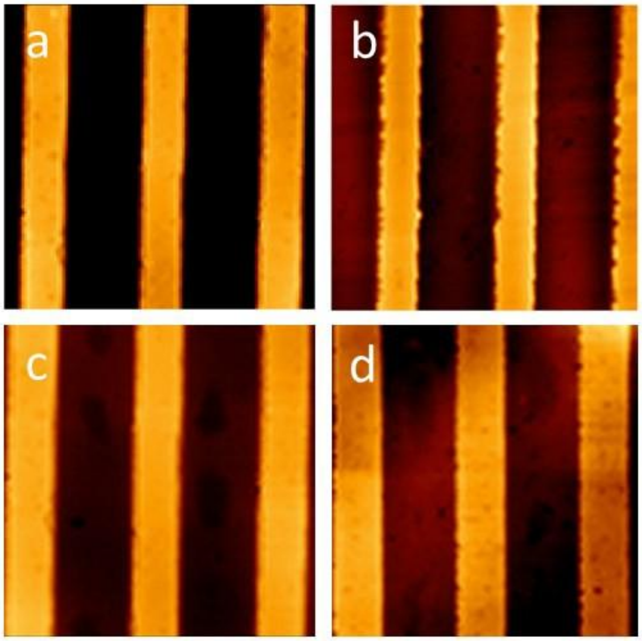

Figure S3: AFM imaging showing the micropatterned topography of SBS films, for non-doped samples (a), samples doped with 0.01% BTNP (b), samples doped with 0.1% BTNP (c), and samples doped with SBS+1% BTNP (d). The scan size of all the images is 50 µm x 50 µm.

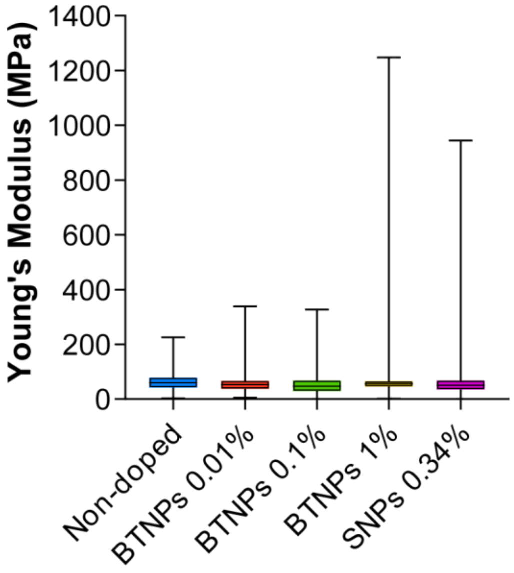

Figure S4: Young's modulus of SBS films with different BTNP concentrations and SNP, derived from AFM nanoindenting analyses. Differently from the graph reported in the main manuscript, here all the data are shown, including the outliers.

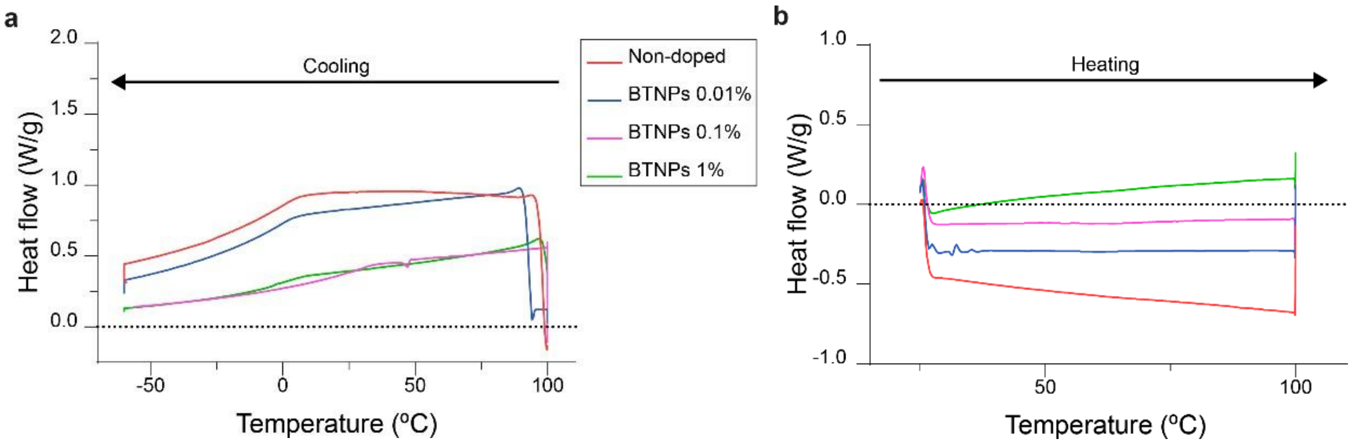

Figure S5: Thermal characterization of SBS films with different BTNP concentrations. (a) Cooling flow curve; (b) heating flow curve.

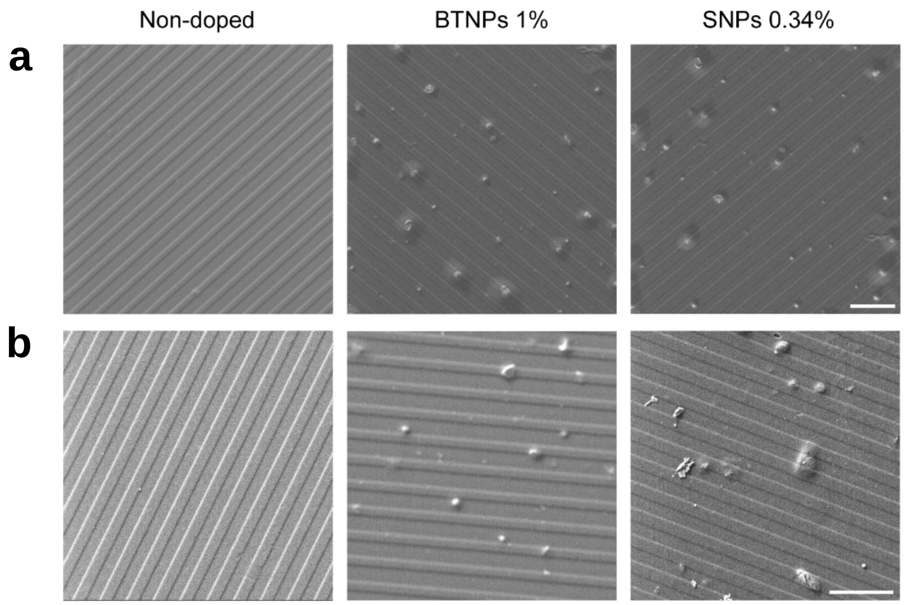

Figure S6: SEM images for SBS films with and without 1% BTNPs and 0.34% SNPs. Images taken before (a) and after 10 days in cell culture conditions (b). Magnification: 880x (a) and 1250x (b), both scale bars = 50 µm.

	**Non-doped**	**BTNPs 1%**	**SNPs 0.34%**
Day 0	9.63 ± 0.77	9.58 ± 0.66	9.75 ± 0.55
Day 10	9.60 ± 0.91	10.02 ± 0.64	9.31 ± 0.64

Table S3: Grooves' width before and after being maintained for ten days in cell culture conditions. Three measures were done on at least 10 channels per sample, for 3 samples. Data are expressed in µm, as mean ± sd. Between all groups there is not a significant difference in groove width between day 0 and day 10 (two-way ANOVA with Tukey's post hoc test, $p>0.05$ for each group).

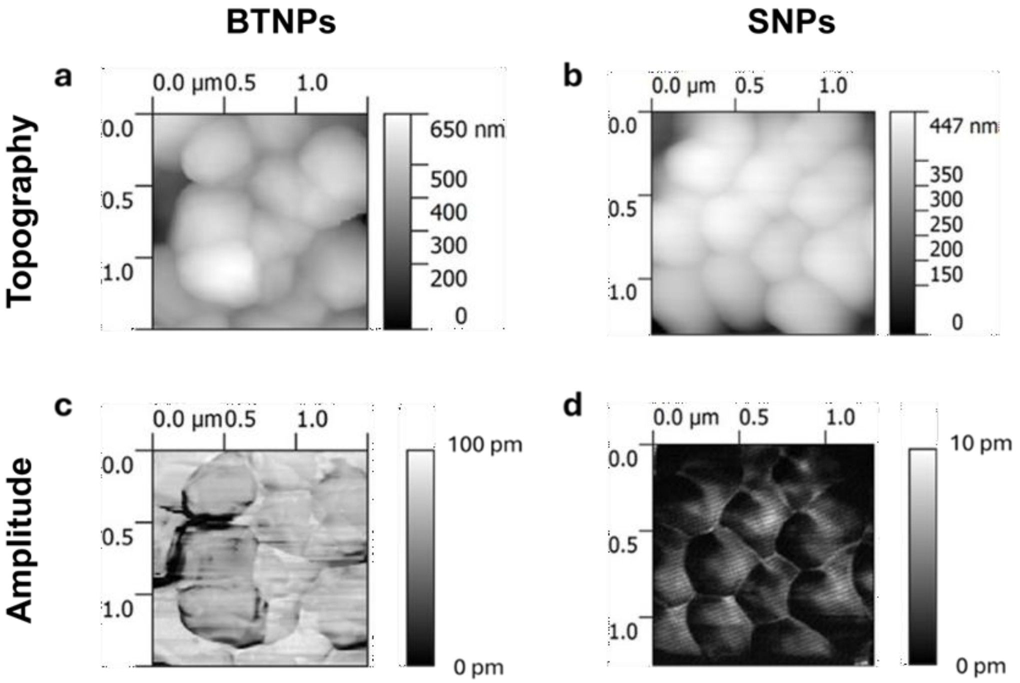

Figure S7: Representative images of the topography of BTNPs (a) and SNPs (b), and of their response in terms of amplitude of deformation as a response to the application of an alternating voltage of 1 V, (c) BTNPs and (d) SNPs. Particles were dropped onto a conductive substrate made of indium tin oxide before the measurement.

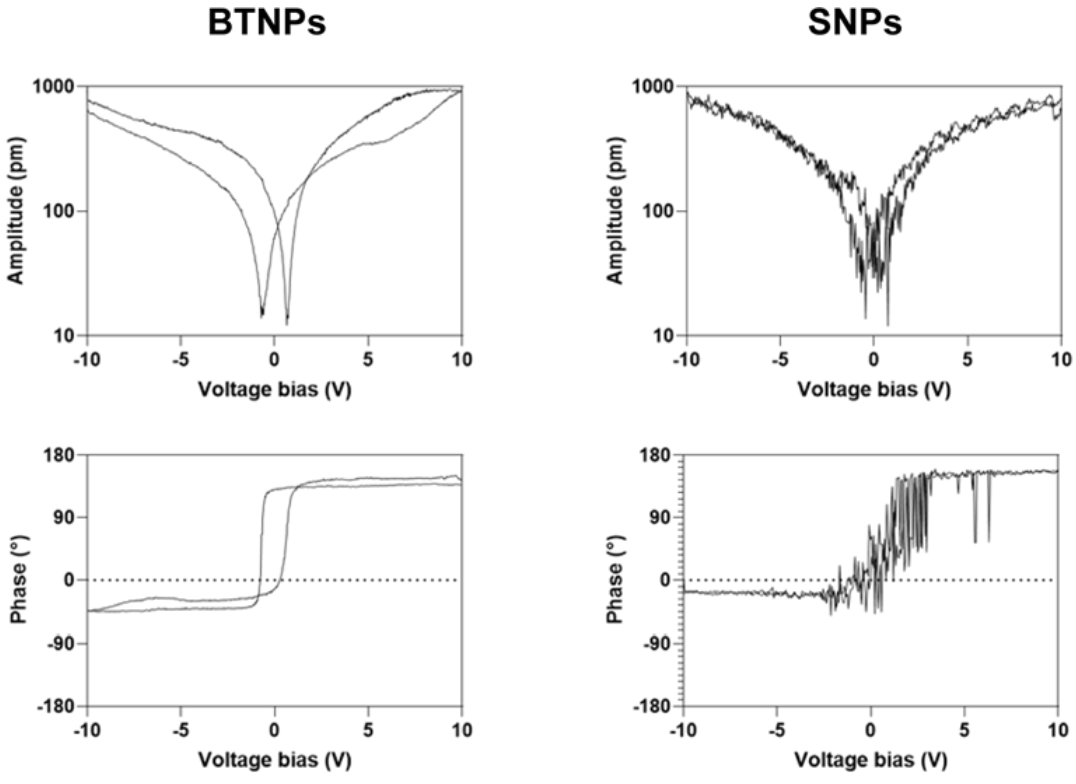

Figure S8: Comparison between the amplitude (top) and phase (bottom) signals of for silica (SNPs) and barium titanate (BTNPs) nanoparticles.

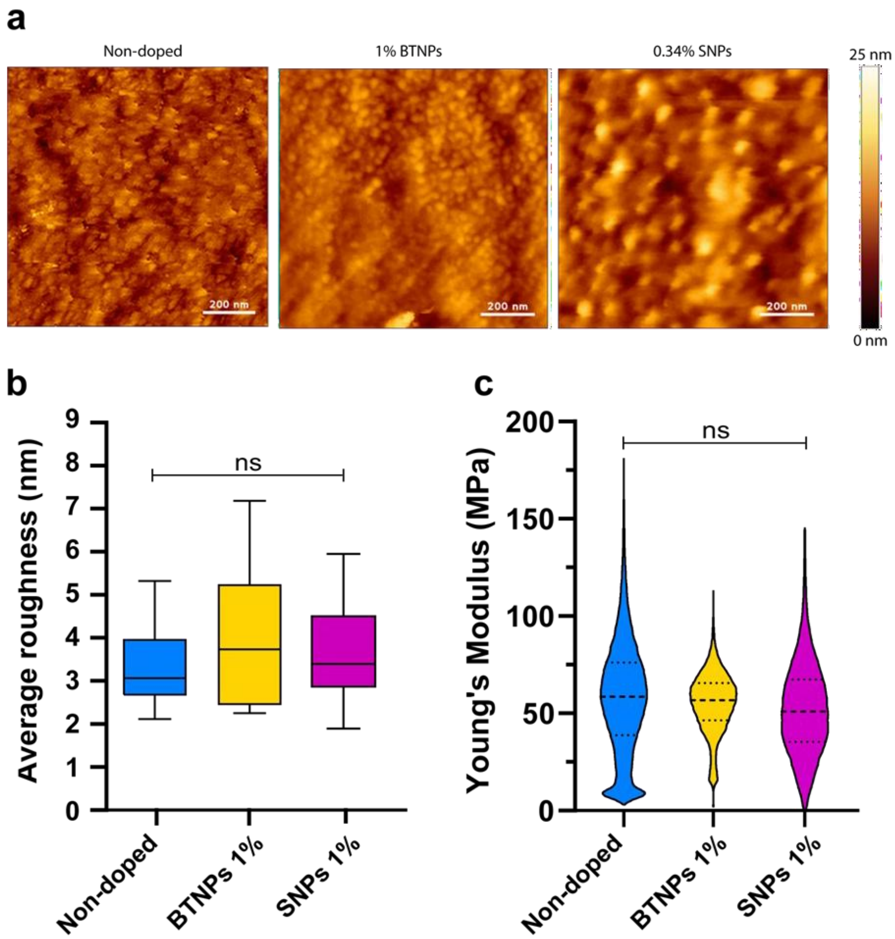

Figure S9: AFM characterization of the doped SBS films. (a) Representative high-magnification AFM images acquired on the surface SBS films 1% BTNP and 0.34% SNP. (b) average roughness of the thin films. A minimum of 45 areas have been analyzed, per each sample type. (c) Young's modulus of SBS films with different BTNP concentrations, data filtered for outliers.

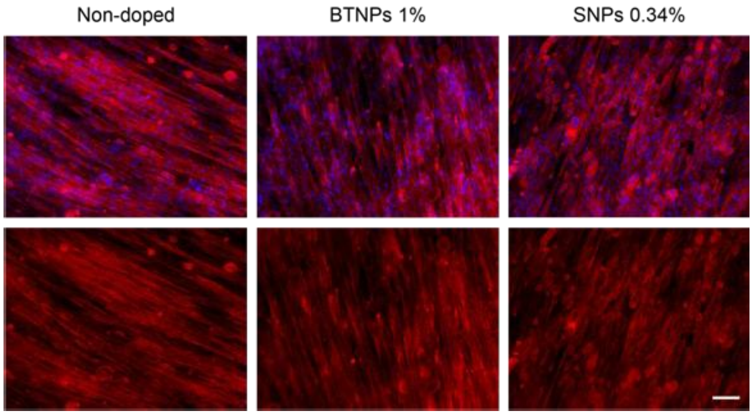

Figure S10: Evaluation of myotube formation on SBS films with different BTNP concentrations. Immunofluorescence images show F-actin (in red) and nuclei (in blue), on the 6th day in differentiation medium (DM6). Scale bar = 100 µm.

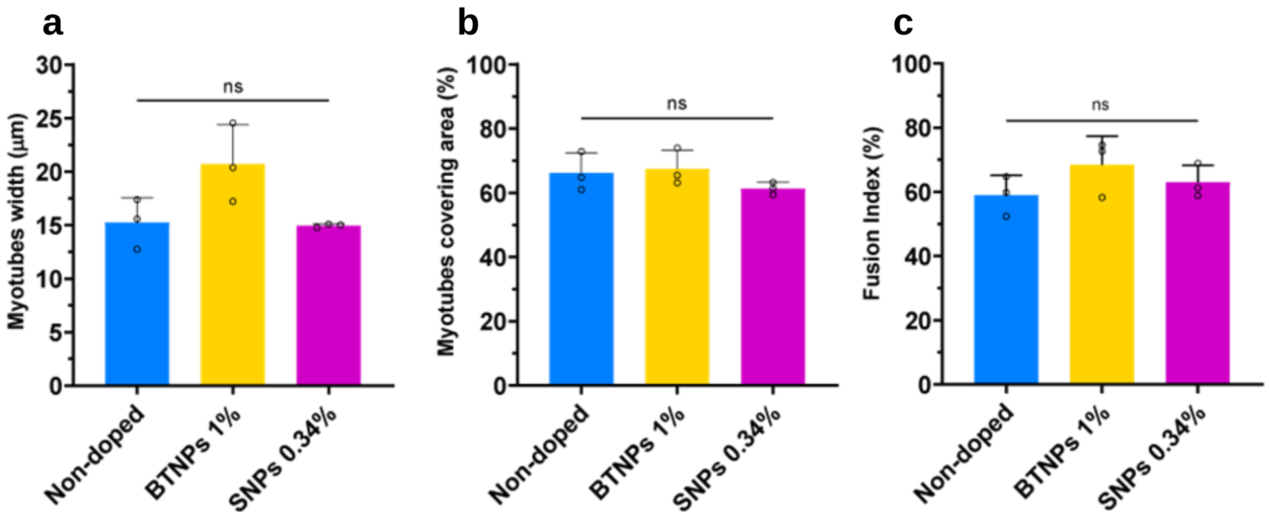

Figure S11: Quantitative myotube analysis derived from immunofluorescence images. (a) Myotube width; (b) area covered by myotubes; (c) fusion index, on SBS films with different BTNP concentrations. Overlaid scattered dots in each experimental group represent biological replicates (n = 3), each derived from the average of three technical replicates.

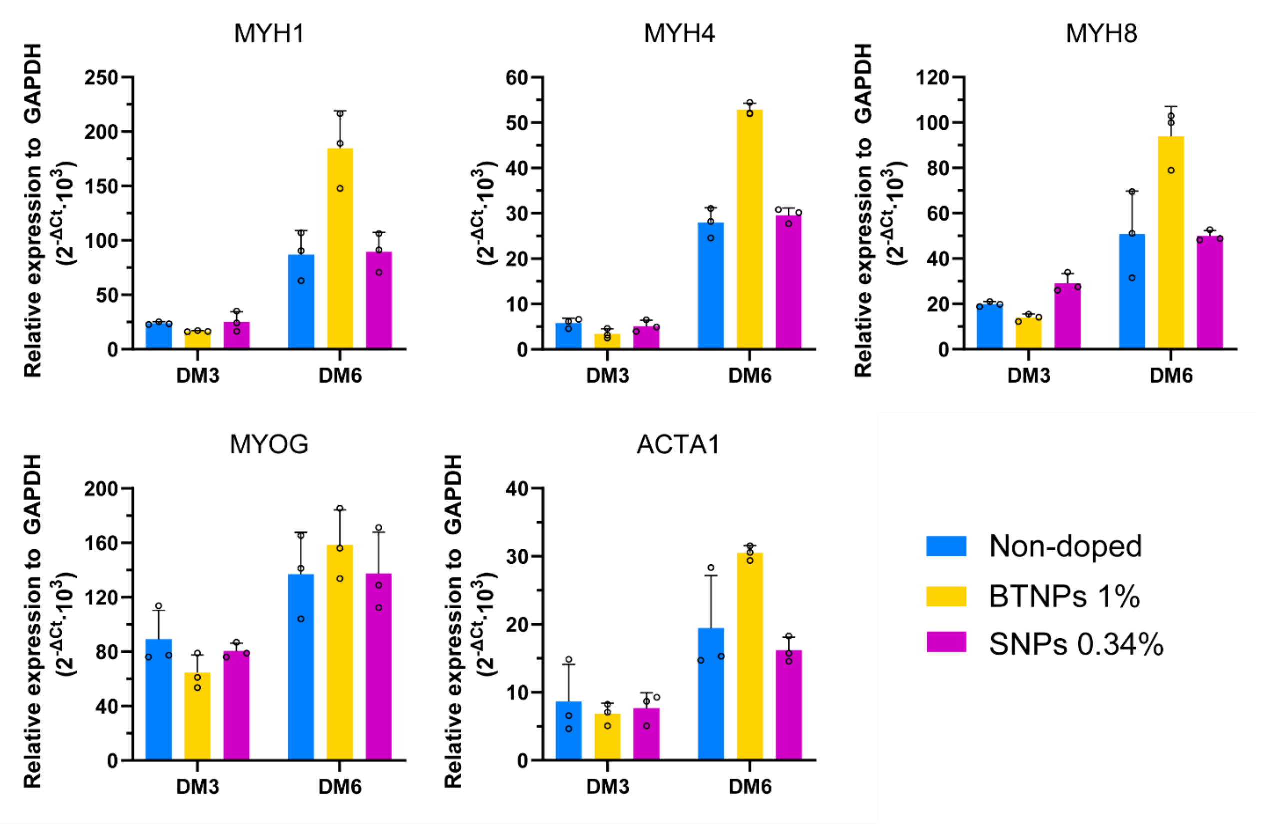

*Figure S12: Gene expression analyses on myoblasts differentiated on non-doped and doped films, on day 3 and 6 of differentiation. DM: differentiation medium. *=p<0.05, **=p<0.01, ***=p<0.001, ****=p<0.0001. Overlaid scattered dots in each experimental group represent biological replicates (n = 3), each derived from the average of three technical replicates.*

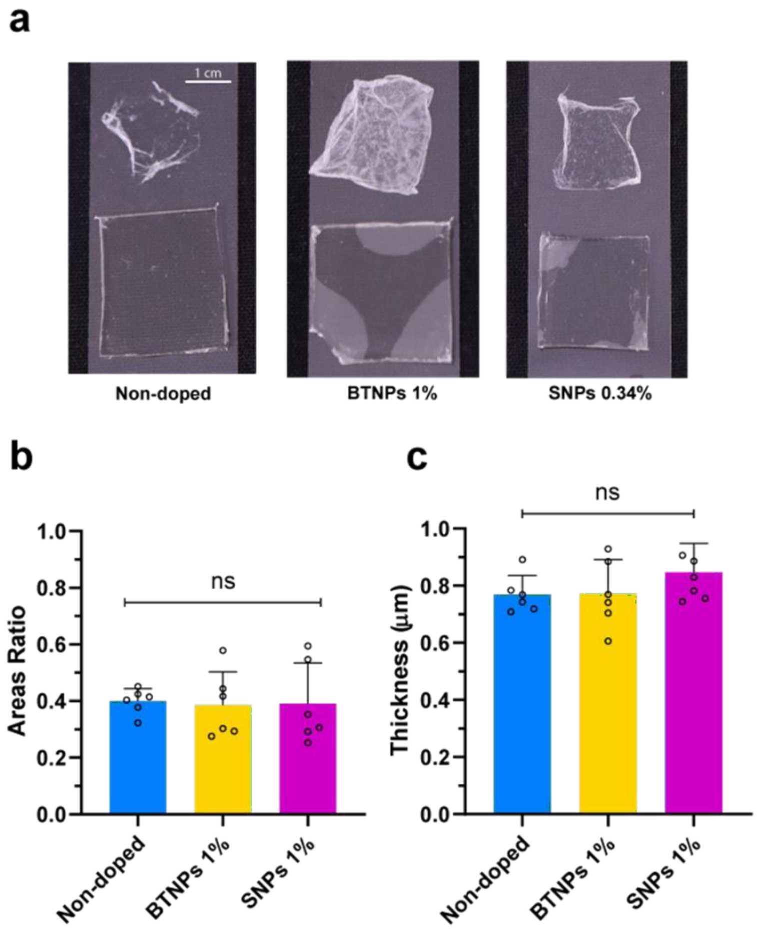

Figure S13: Characterization of doped films in terms of thickness and manipulability. (a) Images of films (SBS 40 mg/mL) non-doped and doped with 1% BTNPs or 0.34% SNPs, after PVA dissolution (scale bar 1 cm); (b) film/mold area ratio to quantify manipulability. (c) Film thickness; overlaid scattered dots in each experimental group represent biological replicates (n = 6), each derived from the average of three different measures per film.
